# Prenatal Exposure to a Polychlorinated Biphenyl (PCB) Congener Influences Fixation Duration on Biological Motion at 4-Months-Old: A Preliminary Study

**DOI:** 10.1371/journal.pone.0059196

**Published:** 2013-03-28

**Authors:** Hirokazu Doi, Shota Nishitani, Takashi X. Fujisawa, Tomoko Nagai, Masaki Kakeyama, Takahiro Maeda, Kazuyuki Shinohara

**Affiliations:** 1 Department of Neurobiology and Behavior, Graduate School of Biomedical Sciences, Nagasaki University, Nagasaki, Japan; 2 Center for Disease Biology and Integrative Medicine, Graduate School and Faculty of Medicine, The University of Tokyo, Tokyo, Japan; 3 Department of Island and Community Medicine, Island Medical Research Institute, Graduate School of Biomedical Sciences, Nagasaki University, Nagasaki, Japan; Indian Institute of Toxicology Research, India

## Abstract

Adverse effects of prenatal exposure to polychlorinated biphenyl (PCB) congeners on postnatal brain development have been reported in a number of previous studies. However, few studies have examined the effects of prenatal PCB exposure on early social development. The present study sought to increase understanding of the neurotoxicity of PCBs by examining the relationship between PCB congener concentrations in umbilical cord blood and fixation patterns when observing upright and inverted biological motion (BM) at four-months after birth. The development of the ability to recognize BM stimuli is considered a hallmark of socio-cognitive development. The results revealed a link between dioxin-like PCB #118 concentration and fixation pattern. Specifically, four-month-olds with a low-level of prenatal exposure to PCB #118 exhibited a preference for the upright BM over inverted BM, whereas those with a relatively high-level of exposure did not. This finding supports the proposal that prenatal PCB exposure impairs the development of social functioning, and indicates the importance of congener-specific analysis in the risk analysis of the adverse effects of PCB exposure on the brain development.

## Introduction

Polychlorinated biphenyls (PCBs) are environmental pollutants with well-known toxic effects on biological organisms. Although the production of PCBs has been strictly regulated in most industrialized countries since the late 1970 s [1], they are still prevalent in the environment due to their high persistence and resistance to bio-degradation [1], [2], [3]. Exposure to PCBs is known to have a wide-range of adverse effects on human physiological functioning, such as interference in immune function [4], thyroid hormone metabolism [5], and sex-hormone homeostasis [6], [7]. These adverse effects were traditionally thought to be caused by coplanar dioxin-like PCBs via pathways mediated by the aryl-hydrocarbon receptor [8], [9], but some studies have indicated the contribution of non dioxin-like PCB congeners [10], [11]. Thus, little is known about the mechanisms by which PCB toxicity affects human bodily function.

PCBs accumulate in the food chain, and human adults are primarily exposed to them via daily food consumption [12]. In addition, PCBs are known to cross the placental barrier [13], [14]. As such, fetuses can be exposed to PCBs in the prenatal period, during which the development of the neural system is highly vulnerable to exogenous factors. In addition, estrogen has been found to alter the concentration of dopamine in the brain [15], [16], as well as synaptic patterning [17], and neuronal apoptosis [18]. On the basis of these findings, it has been proposed that PCBs may impair the functional and organizational processes of neural structures through interference with sex-hormone activity. Thus, it is possible that brain development is adversely affected by prenatal exposure to PCBs. Consistent with this notion, longitudinal studies conducted in Holland, Germany, the United States and Japan have revealed an inverse relationship between the level of prenatal PCB exposure and neurobehavioral function during each developmental stage [1], [19]–[29] (see [2], for a review).

Previous studies on neurotoxicity of PCBs have typically examined brain development using standardized test batteries of psychomotor and intelligence tests, such as the Bayley Scale of Infant Development, the Fagan Test of Infant Intelligence, the Kaufman Assessment Battery for Children and the Raven Progressive Matrices Test [3], [25], [30]. These test batteries enable researchers to measure various aspects of brain function, including spatial cognition, language acquisition, and memory capacity in a relatively short time. However, these batteries do not include tests of a child's ability in several important domains of brain function, so cannot provide a comprehensive understanding of the effects of prenatal PCB exposure on neural development.

Among the cognitive functions that have been largely neglected in previous studies of the neurotoxicity of PCBs, the development of socio-cognitive function is particularly important. The term “socio-cognitive function” collectively refers to a diverse array of social functions, such as recognition of emotional signals and attentiveness towards social entities [31]–[33] (for a review, see [34]). The development of these abilities enables infants to establish affective relationships with others, and increases their knowledge about the environment. Given the importance of social cognition in early human development, the investigation of prenatal PCB exposure on the development of socio-cognitive functioning requires special attention in risk analysis of prenatal exposure to PCB congeners. However, few attempts have been made to clarify this issue.

The present study aimed to elucidate this question by examining the effects of prenatal PCB exposure on the early development of socio-cognitive function. Importantly, we sought to establish a reliable measure of infant socio-cognitive function, by measuring preferential looking patterns towards biological motion (BM) stimuli [35] as an index of socio-cognitive ability, and examined the link between PCB levels in the umbilical cord blood serum and preferential looking patterns to upright and inverted BM stimuli in four-month olds.

**Figure 1:**
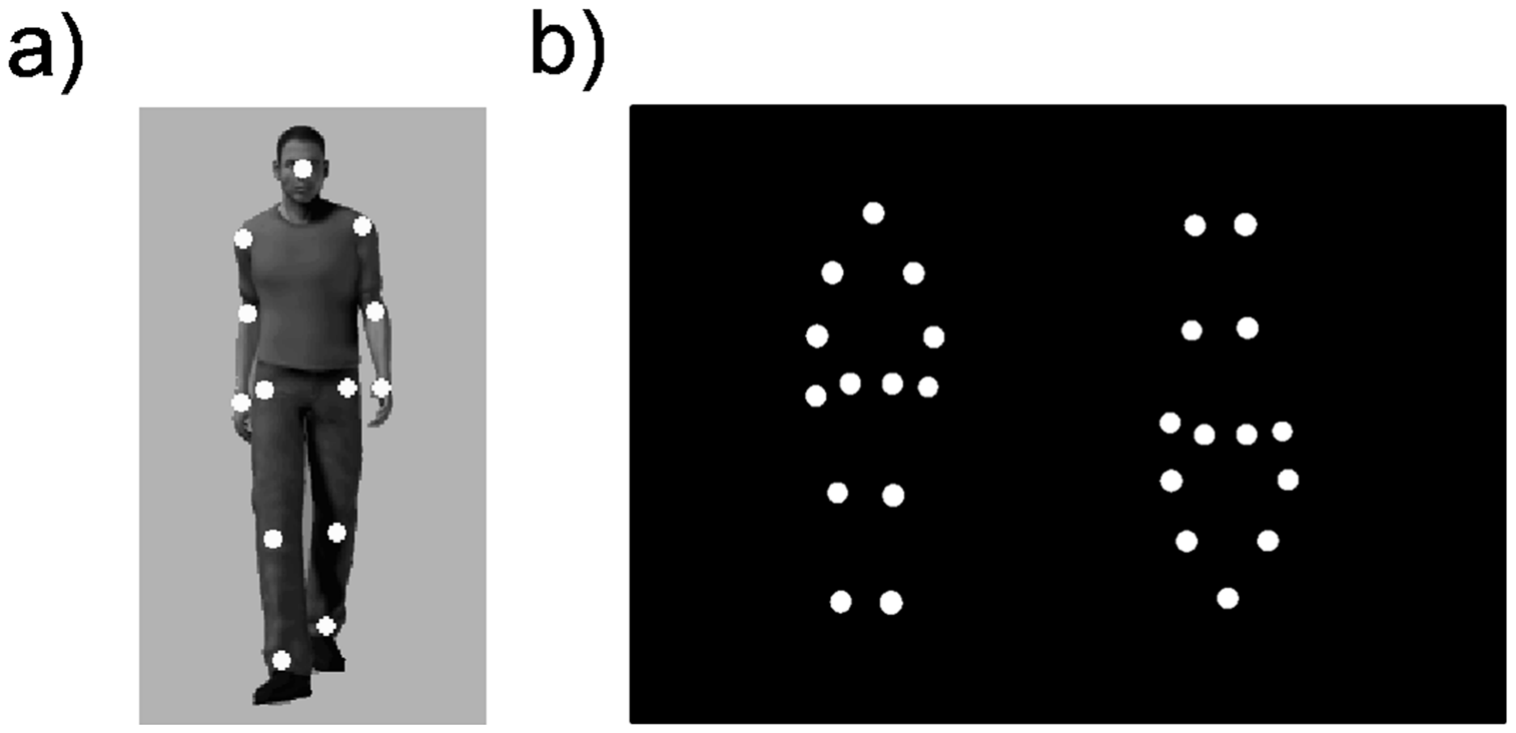
Examples of point-light display and stimulus display. Panel a) The schematic depiction of the concept of point-light display. The bodily motion of a human walker is represented as a constellation of moving white dots attached to each joint. Panel b) An example of the stimulus display in preferential looking experiment.

BM stimuli typically consist of animated moving point-lights depicting human bodily movement. In a seminal study of BM perception, Johansson [35] video-taped motion paths of luminescent markers attached to each joint of a human body, and depicted human bodily movement as a constellation of moving white dots, referred to as a point-light display as schematically depicted in Figure 1–a). Interestingly, later studies reported that normal adults and children can recognize various kinds of information, such as emotional states [36]–[38] and the model's gender [38]–[40], from a BM point-light display, within a relatively short duration [41]. In addition, the vivid impression of humanness can be effectively eliminated by inverting BM stimuli in the vertical orientation [42]–[44].

The previous studies have reported that infants prefer to look at upright BM stimuli compared with vertically inverted BM stimuli as early as two days after birth [45], in accord with the notion that the human neural system is innately tuned to detect human bodily movement. In addition, a series of studies by Klin et al reported abnormal fixation patterns and preferences for upright and inverted BM stimuli in infants with high risk of autism [46], [47], a developmental disorder characterized by the severe deficiencies in social communication skills including the deficiency in BM perception [48]. Thus, deficiencies in BM processing in early development may constitute a hallmark symptom of socio-cognitive development [46], [47], [49], [50].

## Materials and Methods

### 2.1. Ethical Statement

The preset study has been conducted according to the principles expressed in the Declaration of Helsinki. The protocol of the present study was approved by the ethical committee of the Graduate School of Biomedical Sciences, Nagasaki University. The parents of the infants have given written informed consent for participation.

### 2.2. Participants

A total of 40 pairs of healthy term infants (23 boys and 17 girls) and their mothers participated in the present study. Data from 11 pairs of participants were discarded from the final analysis because of poor data quality. The data from these participants were discarded from the dataset mainly due to failures in fixation duration measurement. The causes of these failures included fussiness, excessive movements, pointing to the stimuli by the mother, and exclusive fixation on the left or right side of the display. The detailed characteristics of the infants and mothers included in the final analysis are summarized in Table 1.

**Table 1:** Characteristics of infants and mothers included in the dataset used for the statistical analysis.

		N	%
Infant Sex	Male	11	37.9
	Female	18	62.1
Parity	0	7	24.1
	1	22	75.9
Infant's age (days)		131.3 (14.6)	
Maternal age (years)		30.9 (4.06)	
Smoking	Yes	5	17.2
	No	22	75.9
	Missing	2	6.9
Feeding Type	Breast	5	17.2
	Mixed	16	55.2
	Formula	2	6.9
	Missing	6	20.7

Standard deviation shown in parentheses. Maternal age indicates the age of mother at the point of delivery. Breast: Exclusively breast-fed, Mixed: Mixed use of breast- and formula-feeding, Formula: Exclusively formula-fed. “Missing” indicates that the data were not available.

### 2.3. Stimuli and Materials

The stimuli were generated on a 17-inch color monitor, viewed binocularly from a distance of approximately 15 cm. Each stimulus consisted of upright or inverted point-light displays of a walking human figure. Point-light human figure displays were created based on three-dimensional coordinate files taken from a database produced by Vanrie and Verfaillie [51]. The upright walking figure was facing towards the viewer, and the inverted BM stimuli were created by inverting the upright BM stimuli in the vertical orientation. Each BM stimulus subtended approximately 5.1 cm in width and 13.5 cm in height at the maximum. One cycle of the walking sequence is constituted by 60 frames and lasted for 1 sec. The initial frame of walking cycle was randomly determined.

A CCD camera was situated 2 cm below the lower edge of the monitor. The images captured by this camera were relayed to the another monitor observed by the experimenter. The experimenter controlled the stimulus presentation using a PC while observing the participants' behavior. The monitor viewed by the experimenter and the PC for controlling stimulus presentation was concealed from the infant's visual field by a partition.

### 2.4. Procedure

#### 2.4.1. Preferential Looking Experiment

The fixation durations on the upright and inverted BM stimuli were measured with an infant-controlled preferential looking procedure. Infants sat on their mother's lap in front of the monitor. First, a geometric figure was presented on the monitor, accompanied by a brief sound effect to attract the infant's attention. The geometrical figure subtended approximately 5.6 cm in height and 5.6 cm in width, and oscillated in the vertical direction. When the experimenter judged that the infant was looking at the monitor, the stimulus presentation was started. If the infant was not looking at the monitor, a new geometric figure was presented as the attention-attracting stimulus. This procedure was repeated until the geometrical figure successfully captured infant's attention.

At the start of the stimulus presentation, a 2.7 cm×2.7 cm white fixation cross flickered at the center of the screen at 10 Hz for 500 ms so that the infant fixated on the center of the monitor. As soon as the fixation cross disappeared, upright and inverted BM stimuli were presented simultaneously. An example stimulus is shown in Figure 1–b). The intervening space between the innermost edges of BM stimuli was roughly 7 cm. The BM stimuli were presented on the monitor until the infant looked away from the monitor for about 20 s consecutively (for a similar procedure, see, [52]). After brief intermission, the next trial was conducted. The second trial was essentially the same as the first one with the sole exception that the locations of the upright and inverted BM stimuli were flipped. The side of the upright and inverted BM stimuli in the first trial was counterbalanced across participants.

Fixation behavior during the trials was captured using a CCD camera and recorded on a hard disk. The recorded video was analyzed off-line in a frame-by-frame manner to quantify the fixation durations for upright and inverted BM stimuli.

#### 2.4.2. PCB Congener Concentration Measurement

Umbilical cord blood samples (<10 mL) were collected immediately after birth using a standard procedure, which included careful cleansing of the cord and strict puncture of the umbilical vein to avoid maternal contamination. The concentrations of 17 PCB congeners (PCB IUPAC #74, #99, #118, #126, #138, #146, #153, #156, #163, #170, #177, #178, #180, #183, #187, #194, #199) in the umbilical cord blood serum samples were measured using high resolution gas-chromatography. The measurements of the PCB congener concentrations were conducted by Environmental Risk Research Center, IDEA Consultants, Inc. It has previously been shown that the sum concentration of these 17 congeners typically accounts for approximately 80% of the total PCB concentration in cord blood samples [53].

#### 2.4.3. Neurobehavioral Assessment

To examine the effect of neurobehavioral development on infant's preferential looking patterns, the infants' development was assessed using the KIDS questionnaire. The KIDS questionnaire assesses development indices in six domains of neurobehavioral functioning: physical motor, manipulation, receptive language, expressive language, social relations with adults, and feeding [54], [55]. The KIDS questionnaire had been standardized on the basis of the data collected from a large cohort of Japanese children, and is reported to have an acceptable level of reliability [54], [55].

## Results

### 3.1. Relation between PCB Congeners and Preferential Looking Pattern

The fixation durations for upright and inverted BM stimuli and the concentrations of 17 PCB congeners for each participant are listed in Table 2. To examine the link between the concentrations of PCB congeners and preferential looking pattern, fixation durations in each condition were entered as dependent variables into stepwise regression analyses with PCB congener concentrations as independent variables. The criteria of variable entry and omission were *F*-values corresponding to *p*-values of .05 and .10, respectively. Stepwise regression analysis revealed that the concentration of PCB #118 was an independent predictor of the fixation duration on the inverted BM, *F* (1, 27)  = 7.99, *p*<.01, *β* = .48. There was no independent predictor of the fixation duration for upright BM.

**Table 2:** The Fixation Duration in each condition and the concentrations of PCB congeners.

		Fixation Duration (sec)			PCB Concentration (pg/g-wet)																
Group	Subject	Inverted	Upright	Total	#74	#99	#118	#126	#138	#146	#153	#156	#163	#170	#177	#178	#180	#183	#187	#194	#199
Low	1	16.2	32.1	48.3	1.5	1.2	2.5	N.D	4.6	1.7	10	1.4	4.6	3.3	0.7	0.8	8.3	0.8	4.5	1.5	1.3
Low	2	11.1	5.8	16.8	1.4	1.4	2.2	N.D	4.2	1.9	12	1.4	4.1	3.7	0.6	0.9	9.4	0.6	3.9	1.0	1.3
Low	3	4.7	30.3	35.0	0.9	0.8	1.3	N.D	2.6	1.5	7	1.1	2.9	1.9	0.8	0.6	6.1	0.6	3.0	0.6	0.8
High	4	10.1	19.5	29.6	1.7	2.6	4.7	N.D	6.7	3.3	19	1.6	5.4	4.1	1.4	0.8	11.0	1.1	5.2	1.8	1.8
Low	5	35.6	44.3	80.0	2.1	1.7	3.6	N.D	5.4	2.1	17	1.6	4.4	3.0	0.9	0.8	11.0	1.0	4.8	1.3	1.3
High	6	49.8	55.7	105.5	3.3	4.7	8.4	N.D	13	6.6	39	2.6	11.0	7.5	2.3	2.2	24.0	2.2	12	3.4	3.6
High	7	14.6	20.5	35.1	1.8	3.3	4.4	N.D	6.6	2.4	17	1.0	3.0	2.1	0.8	0.7	5.9	1.2	3.9	0.7	0.8
High	8	18.5	18.0	36.5	1.7	2.9	3.9	N.D	8.5	4.0	27	1.7	4.9	3.7	1.3	1.1	11.0	1.3	6.0	1.5	1.8
High	9	37.3	20.4	57.7	3.0	2.9	4.6	N.D	8.0	4.0	25	1.7	6.3	3.3	1.5	0.9	11.0	1.1	6.1	1.2	1.3
High	10	14.2	20.9	35.1	7.3	4.9	7.2	N.D	17	6.7	41	3.7	13	8.4	2.9	2.5	23.0	2.5	12.0	2.4	3.1
High	11	33.1	24.2	57.3	2.2	1.7	4.1	N.D	5.8	2.3	14	1.2	3.8	3.3	0.8	0.7	6.4	1.0	3.6	0.9	0.9
Low	12	10.3	8.9	19.2	1.9	2.6	2.9	N.D	5.5	2.3	15	0.8	3.5	3.3	1.0	0.8	7.8	1.0	5.2	0.9	1.2
High	13	63.2	47.0	110.3	2.4	3.1	5.2	N.D	8.9	3.8	24	1.6	5.9	4.7	1.6	1.3	12.0	1.6	6.9	1.5	1.7
Low	14	14.1	19.7	33.8	1.8	1.1	1.5	N.D	3.1	1.2	9	0.8	2.6	2.5	0.8	0.8	6.0	0.7	2.9	0.9	0.9
Low	15	15.9	62.6	78.5	2.2	2.4	3.2	N.D	7.1	2.4	19	1.5	4.1	3.9	1.0	0.9	9.6	1.2	5.5	1.5	1.4
High	16	15.0	9.0	24.0	2.8	3.1	5.5	N.D	7.8	3.8	24	1.6	6.9	5.3	1.6	1.6	15.0	1.7	7.9	1.8	2.2
High	17	29.1	10.2	39.4	4.1	1.6	3.8	N.D	6.0	2.7	18	1.8	4.2	3.8	1.0	1.0	11.0	1.2	4.9	1.5	1.5
Low	18	15.9	60.2	76.1	2.2	1.7	3.2	N.D	3.9	2.2	12	0.9	3.1	2.6	0.9	0.6	5.8	0.8	3.5	0.8	0.9
Low	19	20.6	23.9	44.5	1.5	1.2	2.5	N.D	4.6	1.7	10	1.4	4.6	3.4	0.7	0.8	8.3	0.8	4.6	1.5	1.3
Low	20	24.1	25.7	49.7	1.5	1.4	2.3	N.D	4.3	1.9	12	1.5	4.3	3.8	0.7	0.9	9.7	0.6	4.0	1.0	1.4
Low	21	2.8	25.3	28.1	1.0	0.8	1.4	N.D	2.8	1.6	8	1.2	3.1	2.0	0.8	0.7	6.4	0.7	3.1	0.7	0.9
High	22	24.6	22.6	47.3	1.7	2.5	4.6	N.D	6.6	3.3	19	1.6	5.3	4.0	1.3	0.8	11.0	1.1	5.1	1.8	1.8
Low	23	31.7	15.0	46.7	2.1	1.7	3.6	N.D	5.4	2.1	17	1.6	4.4	3.0	0.9	0.8	11.0	1.0	4.8	1.3	1.3
High	24	18.0	20.2	38.2	1.7	3.2	4.3	N.D	6.4	2.3	16	0.9	2.9	2.0	0.8	0.7	5.7	1.2	3.8	0.7	0.7
High	25	6.8	9.9	16.7	1.8	2.9	4.0	N.D	8.6	4.0	27	1.7	5.0	3.7	1.3	1.1	11.0	1.3	6.0	1.5	1.8
High	26	35.4	14.2	49.6	1.7	2.1	4.7	N.D	6.7	2.5	13	1.4	4.1	4.9	1.2	1.0	8.2	1.2	4.2	1.9	2.4
High	27	15.9	11.1	27.0	2.2	3.1	7.0	N.D	11.0	3.8	21	1.6	6.6	5.7	1.9	1.4	13.0	1.8	6.8	2.1	2.3
Low	28	1.1	0.3	1.4	1.4	1.8	2.5	N.D	5.8	1.7	13	1.1	3.4	2.7	0.7	0.6	6.6	0.7	3.7	1.0	1.2
Low	29	11.9	9.7	21.6	1.3	1.8	2.8	N.D	5.2	1.6	10	0.9	3.4	3.4	0.9	0.5	6.3	1.1	3.5	0.8	1.2
	M	20.7	23.7	44.4	2.1	2.3	3.9		6.6	2.8	17.8	1.5	4.9	3.8	1.1	1.0	10.1	1.1	5.2	1.4	1.5
	SD	14.1	16.2	25.5	1.2	1.0	1.7		3.1	1.4	8.3	0.6	2.3	1.5	0.5	0.5	4.5	0.5	5.2	0.6	0.7

M: Mean, SD: Standard Deviation N.D.: None Detected.

The fixation durations are rounded at the first decimal place.

To further elucidate the effects of PCB #118 concentration on preferential looking pattern, the participants were split into two groups, namely, a High Exposure Group and a Low Exposure Group, based on the median PCB #118 concentration value. The participant with the median concentration (No. 17) was included in the High Exposure Group, because the participant (No. 8) exhibiting PCB #118 concentration next closest to the median value was in this group. To be more specific, participant No.17 had the median PCB #118 concentration. Because the dataset had odd number of participants (N = 29), the remaining 28 participants were split into two groups, *i.e.* those with PCB #118 concentration higher/lower than the median value classified into High/Low Exposure Group, respectively. According to this method of grouping, the participant (No. 8), who had PCB #118 concentration closest to No. 17, was classified into High Exposure Group. Therefore, we decided to include No. 17 also in High Exposure Group.

**Figure 2:**
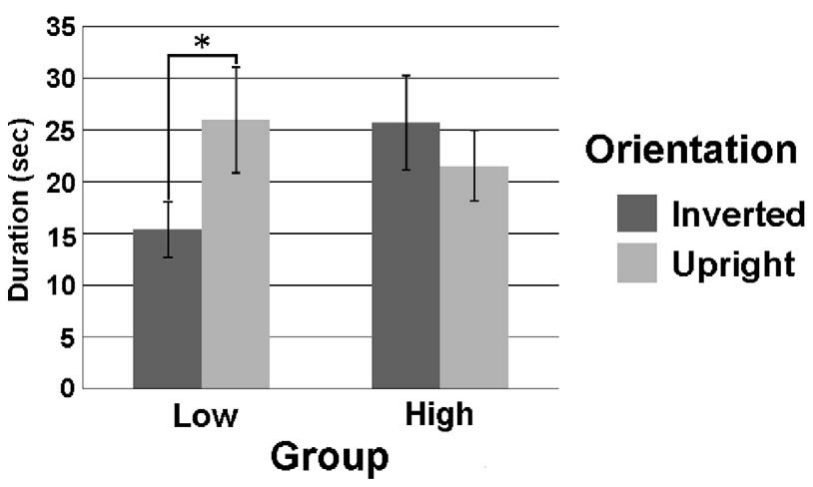
Averaged fixation durations in each condition. Error bars indicate standard errors. *p<.05, for the simple main effect of Orientation.

The characteristics of the High and Low Exposure Groups are summarized in Table 3. The average fixation durations in the Upright and Inverted BM conditions in each Exposure Group are shown in Figure 2, together with standard errors. The fixation durations were entered into a two-way analysis of variance (ANOVA) with Exposure Group (High-Low) as a between-participants factor and Orientation (Upright-Inverted) as a within-participants factor. We found no significant main effect of either Exposure Group, *F* (1, 27)  = 0.35, *p*>.10, or Orientation, *F* (1, 27)  = 1.36, *p*>.10. However, the interaction between Exposure Group and Orientation was significant, *F* (1, 27)  = 7.16, *p*<.05.

**Table 3:** Characteristics of High and Low Exposure Groups.

		Low	High
Infant Sex	Male	10	7
	Female	4	8
Infant's age (days)		131.5 (9.4)	131.1 (18.8)
PCB #118 (pg/g-wet)		2.54 (0.76)	5.09 (1.37)

Standard deviations shown in parentheses.

To clarify the source of the interaction, we conducted simple main effect analyses. The analyses revealed a significant simple main effect of Orientation in the Low, *F* (1, 27)  = 7.34, *p*<.05, but not the High Exposure Group, *F* (1, 27)  = 1.14, *p*>.10. The simple main effect of Exposure Group did not reach significance either in the Inverted, *F* (1, 54)  = 3.45, *p*>.05, or the Upright Orientation condition, *F* (1, 54)  = 0.59, *p*>.10.

### 3.2. Relations between KIDS Development Indices, PCB Congeners, and Preferential Looking Pattern

We analyzed the relationships between KIDS development indices, PCB congener concentrations and fixation duration. Because development indices were not available for five infants, the following analyses were conducted with the data of the remaining 24 participants.

The correlation coefficients between the KIDS development index in each sub-domain of neurobehavioral function and PCB congeners are summarized in Table 4. Significant correlations were only observed between the development index of expressive language and the concentrations of PCB #156 and PCB #199, indicating that infants exposed to high levels of congeners exhibited significantly delayed development of expressive language.

**Table 4:** Correlation Coefficients between the concentrations of PCB congeners and KIDS Development Indices.

	KIDS Development Index					
PCB IUPAC	Physical Motor	Manipulation	Receptive Language	Expressive Language	Social Relations	Feeding
#74	0.02	0.03	−0.05	0.13	0.34	0.14
#99	−0.04	−0.09	−0.30	−0.002	0.13	0.19
#118	0.02	−0.15	−0.28	−0.09	0.03	0.31
#138	−0.09	−0.27	−0.27	−0.18	−0.08	0.14
#146	0.04	−0.28	−0.27	−0.22	−0.12	0.09
#153	−0.05	−0.28	−0.22	−0.19	−0.02	0.02
#156	−0.09	−0.38	−0.06	−0.42*	−0.27	−0.05
#163	−0.04	−0.24	−0.06	−0.26	−0.12	0.09
#170	−0.16	−0.23	−0.14	−0.37	−0.27	0.17
#177	0.12	−0.21	−0.19	−0.26	−0.09	0.20
#178	−0.08	−0.18	−0.14	−0.36	−0.21	0.16
#180	−0.12	−0.25	−0.07	−0.35	−0.13	0.06
#183	−0.01	−0.16	−0.21	−0.19	0.03	0.23
#187	−0.11	−0.21	−0.08	−0.24	−0.04	0.06
#194	−0.10	−0.22	−0.11	−0.35	−0.25	0.07
#199	−0.13	−0.22	−0.19	−0.48*	−0.35	0.17

* *p*<.05.

Finally, the correlations between the KIDS developmental indices and fixation duration with upright and inverted BMs were examined, but the analyses did not reveal any significant correlations, *Rs^2^* <0.12, *ps* >.05.

## Discussion

The present study examined the effects of prenatal PCB exposure on fixation durations on upright and inverted BM stimuli in four-month-olds. The results revealed that four-month olds with low levels of prenatal exposure to dioxin-like PCB #118 revealed a significant preference for upright BM stimuli over inverted BM stimuli, whereas those with relatively high levels of prenatal PCB exposure exhibited no such preference. Scores on the expressive language development index exhibited significant negative correlations with the concentrations of the non dioxin-like PCB #199 and the dioxin-like PCB #156.

Developmental studies have reported that human infants exhibit a preference for upright over inverted BM stimuli as early as two days after birth [45], suggesting that BM perception is one of the earliest manifestations of sensitivity to socially-meaningful stimuli. This preference for upright BM stimuli is impaired in infants with a high risk of autistic disorders [46], [47], and in developmentally-delayed infants [50]. Taken together with these previous findings, the abnormal preferential looking pattern we observed in four-month-olds with high level of prenatal PCB exposure indicates that prenatal PCB exposure may have adverse effects on early social development. No infants in the present study were exposed to abnormally high-level of PCB congeners. Recently, Endo et al [56] revealed that mice exposed to low dose of dioxin *in utero* show abnormalities in neural regions linked to social-behavior such as amygdala. On the basis of these, it is possible that the adverse effects on the socio-cognitive ability observed in the present study might have partially derived from abnormalities in the neural regions linked to social-behavior, which was caused by prenatal exposure to background-level of PCB congeners.

Two aspects of the current results suggest that the PCB-related abnormality we observed is related to an increased preference for inverted BM among high-exposure infants, rather than a reduced preference for upright BM stimuli. First, the duration of fixation on inverted BM stimuli exhibited a significant positive correlation with PCB #118 concentration, whereas no such relationship was observed for the fixation duration on upright BM stimuli. Second, the fixation duration on inverted BM stimuli tended to be longer in the High Exposure compared to the Low Exposure group, *p*<.10, but *p*>.05,while there was no evidence of a group difference in the fixation duration on upright BM stimuli.

The mechanisms by which prenatal PCB exposure might lead to such an idiosyncratic pattern of preferential looking are currently unclear. In visual cognition studies, the fixation pattern is generally thought to reflect the overt allocation of visuo-spatial attention. Thus, the present finding indicates that infants with high prenatal PCB exposure may be hyper-attentive to visual stimuli with little or no social importance. According to an influential model of visuo-spatial attention allocation [57], objects in the visual scenery compete for attentional resources in the striate and extrastriate visual cortices, and objects selected through this competition process are allocated visual attention. Importantly, socially-important objects are most likely to be allocated attention in typically developed individuals, whereas the processing of the other visual information is inhibited to prevent distracting information from interfering with the processing of important social information. As such, we propose that prenatal exposure to high levels of dioxin-like PCB may impair the inhibitory mechanism that suppresses interference from the distracting socially-unimportant information. Inhibition of distracting information is thought to involve projections from the frontostriatal regions [58] to the visual cortex. Given that dioxin-like PCB are thought to disturb the organization of neural structures [59] through interference with sex-hormone activities [15], [16], [17], [18], we speculatively propose that PCB exposure may impair or at least delay the formation of long-range inhibitory projections from the frontostriatal to occipito-temporal regions. This hypothesis is consistent with the recent finding by White et al [60] that photic stimulation induced greater activation in the bilateral visual association cortex in adolescent boys with high compared with low prenatal PCB/MeHg exposure.

In PCB risk analysis research, it is conventional to sum the levels of several PCB congeners, or to convert PCB concentrations to a single TEQ (Toxic Equivalent Quantity; [9]), as a surrogate index of PCB exposure level. This approach is based on the assumption that dioxin-like PCBs exert concordant influences on biological functioning. However, the present study isolated PCB #118 as a PCB congener that has adverse effects on four-month-olds' preferential looking patterns, while the other dioxin-like PCB congener, PCB #156, exhibited a detrimental effect on the development of expressive language. These results indicate the possibility that different dioxin-like PCB congeners exert different influences on various domains of brain development. Furthermore, the present results also indicate that non dioxin-like PCB #199 might delay the development of the expressive language, which gives support to the contention that non dioxin-like PCB congeners can also exert adverse effects on bodily functions [10], [11].

Overall, the present study investigated the effects of prenatal PCB exposure on the development of socio-cognitive function and developmental indices of neurobehavioral functioning. The results revealed differential influences of PCB congeners on each cognitive and social function, indicating the importance of congener-specific analysis [2] in the risk analysis of the prenatal PCB exposure. Most importantly, the present results show that prenatal exposure to PCB congeners might lead to the postnatal manifestation of the deficiencies in socio-cognitive ability similar to those observed in autistic population [46], [47]. However, as a preliminary experiment, the present study had a small sample size, and the results may have been influenced by several potential confounding variables such as the toxicities of the coeluters. Therefore, the present findings should be replicated with a larger sample size and more comprehensive dataset before drawing definitive conclusions about the effects of prenatal PCB exposure on socio-cognitive development.

One of the potential confounders in the present finding is the type of feeding. Previous studies have shown correlation between levels of prenatal exposure and postnatal lactational exposure to PCBs [61], [62]. According to Lorber and Philips [63], breast-fed infants can accumulate life-time exposure to dioxin-like toxic compounds 3–18% higher than formula-fed infants, which indicates the potential adverse effects of postnatal lactational exposure to PCBs on cognitive development. Consistent with this, a few studies have shown detrimental effect of breast-feeding on cognitive development [64], [65]. Considering that the majority of infants in the present study were breast-fed at some point, one might think that the apparent linkage between prenatal PCB exposure and socio-cognitive development has been mediated by postnatal PCB exposure via breast-feeding. However, a number of longitudinal studies have failed to reveal the detrimental effects of lactational exposure to PCBs [1], [61], [66]–[68], and as such evidence regarding negative effects of postnatal exposure to PCBs via breast-feeding is at best inconclusive. Taking these into consideration, the possibility seems fairly slim that any impairment or delay in socio-cognitive development derive from postnatal lactational exposure to dioxin-like PCB congeners. On the other hand, it is conceivable that the beneficial effects of breast-feeding, possibly attributable to neuro-trophic factors and long-chain polyunsaturated fatty acids contained in breast-milk [1], [61], [68]–[71], have masked subtle adverse effects of PCB congeners other than PCB #118. To address this possibility, it would be interesting to compare the effects of prenatal PCB exposure on socio-cognitive development between exclusively breast-fed and formula-fed infants.
